# Gemtuzumab ozogamicin monotherapy prior to stem cell infusion induces sustained remission in a relapsed acute myeloid leukemia patient after allogeneic stem cell transplantation: A case report

**DOI:** 10.1097/MD.0000000000022064

**Published:** 2020-08-28

**Authors:** Ritsu Sumiyoshi, Haruko Tashiro, Sumiko Saito, Takuji Matsuo, Tadashi Yamamoto, Kensuke Matsumoto, Jun Ooi, Naoki Shirafuji

**Affiliations:** Department of Hematology/Oncology, Teikyo University School of Medicine, Itabashi-ku, Tokyo 173-8606, Japan.

**Keywords:** AML, gemtuzumab ozogamicin, stem cell transplantation, case report

## Abstract

**Rationale:**

Patients with relapsed acute myeloid leukemia (AML) after allogeneic hematopoietic stem cell transplantation (allo-HSCT) have poor prognosis. Many patients are not eligible for 2nd HSCT due to organ dysfunction or other complications that prevent them from tolerating conditioning chemotherapy. In those ineligible patients for 2nd HSCT with myeloablative conditioning regimen, reduced intensity conditioning (RIC) are often used. RIC regimens are less toxic but has a less direct anti-tumor efficacy so that RIC regimens are not suitable for the patients with high tumor burden. To overcome this dilemma, Gemtuzumab Ozogamicin (GO) has been used as a part of RIC regimens to add anti-tumor efficacy. We report here a relapsed AML patient who was treated with GO monotherapy followed by stem cell infusion.

**Patient concerns:**

A 25-year-old male with AML experienced relapse 9 months after allo-HSCT.

**Diagnosis:**

Since he had mild renal and cardiac dysfunction and his AML did not progress rapidly, we decided not to give him an intensive chemotherapy. However, after azacitidine (AZA) and donor lymphocyte infusion therapy, his leukemic blasts did not decrease.

**Interventions:**

Originally, we had planned to proceed with a 2nd allo-HSCT with RIC regimen that consisted of fludarabine, melphalan and fractionated GO (3 mg/m^2^/dose) on day -21, -18, and -15. However, the patient developed appendicitis after the last dose of GO when his neutrophil was 0 cells/μl. Based on his medical acuity, we terminated the rest of the patients conditioning regimen and the patient did not receive any further chemotherapeutics. The patient was still infused with peripheral blood stem cells from the donor on day 0.

**Outcomes:**

His appendicitis was resolved by antibiotics without surgery. His AML has been in CR more than 18 months under AZA maintenance therapy.

**Lessons:**

GO monotherapy could be a conditioning regimen of 2nd allo HSCT from the same donor as the first HSCT for relapsed AML patients.

## 1 Introduction

Patients with relapsed acute myeloid leukemia (AML) after allogeneic hematopoietic stem cell transplantation (allo-HSCT) have poor prognosis. Their 2-year survival rates are less than 20%, independent from the choice of salvage therapy.^[1,2]^ A second transplantation may be the only curative therapy for relapsed AML patients after allo-HSCT. However, AML patients who relapse early after first HSCT or who have organ dysfunctions are frequently intolerable for second HSCT using myeloablative conditioning (MAC) regimen due to regimen related toxicity (RRT). Thus, reduced intensity conditioning (RIC) is often selected for conditioning of a second HSCT. Compared with MAC, RIC has a less RRT, but a less direct anti-tumor effect by conditioning regimens. Because the anti-tumor effect of RIC is weaker than MAC, RIC is not suitable for AML patients with active disease. Therefore, AML patients who experience early relapse after HSCT and refractory or intolerant to re-induction chemotherapy have little indication for second HSCT. Even if the second HSCT was performed to those patients, the prognosis would be dismal. There is a clear need for more effective and less toxic conditioning regimen for 2^nd^ HSCT.

Gemtuzumab ozogamicin (GO) is a chemotherapeutic agent consisting of an anti-CD33 monoclonal antibody linked to calicheamicin. GO has been related to development of sinusoidal obstructive syndrome (SOS), especially when used prior to HSCT,^[3]^ such that using GO for relapsed AML patients after HSCT is challenging. Although initial dosing of GO was 2 rounds of 9 mg/m^2^ intravenously delivered and spaced 14 days apart, recent studies report using lower fractionated doses with less toxicity.^[4,5]^ There are several reports of using GO as a part of conditioning regimens for stem cell transplantation.^[6–8]^

We report here a relapsed AML patient after allo-HSCT who could not receive the planned conditioning regimen but received only GO prior to stem cell infusion. The patient achieved complete remission (CR) on day -1 and has been in CR for more than 18 months.

## 2 Case report

**Figure 1:**
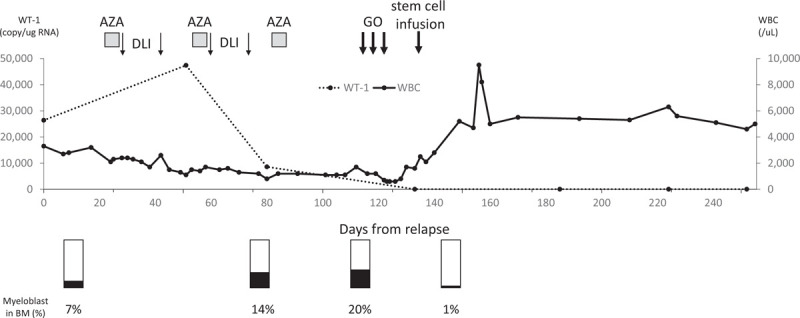
Time course of chemotherapeutic drugs and cell therapy in case 3. The WT-1 copies were determined from peripheral blood samples. The patient was given azacytidine 75 mg/m^2^ for 5 days followed by DLI on day 6 and 13 after AZA with 0.8 × 10^7^/kg and 2 × 10^7^/kg of CD3+ cells from the donor, respectively. Then, he received decreased dose of AZA (37.5 mg/m^2^) for 5 days followed by DLI on day 6 and 13 after AZA with 3 × 10^7^/kg and 5 × 10^7^/kg of CD3+ cells, respectively. The 3rd course of AZA was given as a monotherapy. The copy number of WT-1 was decreased after the 2nd course of AZA. However, 20% of myeloblasts were still in bone marrow after the 3rd course of AZA. WBC = white blood cells; AZA = azacytidine; DLI = donor lymphocyte infusion; GO = ghemtuzumab ozogamicin; PBSCT = peripheral blood stem cell transplantation; BM = bone marrow.

A 25-year-old Japanese male patient who had AML with MLL-ELL gene rearrangement received allogeneic bone marrow transplantation (BMT) from his HLA 7/8 antigen matched sibling donor at his first CR in August 2017. The donor HLA was mismatched by 1 antigen in graft vs host direction. The patient was conditioned with a myeloablative regimen consisting of 12 Gray total body irradiation, 12 g/m^2^ cytarabine, and 120 mg/kg cyclophosphamide. As prophylaxis for acute graft-versus-host disease (GVHD), administration of 3 mg/kg of cyclosporine A (CSP) was started on day -1, and short-term methotrexate was given on days +1, +3, and +6. Since the donor was not a full-match donor, we also added low dose rabbit anti-thymocyte globulin (2.5 mg/kg) on day -4 to prevent GVHD. On day 29, his bone marrow showed complete donor chimerism by short tandem repeats polymorphism (STR) and he remained in CR. He did not develop acute GVHD and CSP was tapered down. However, his AML relapsed 9 months after BMT and he became dependent on platelet transfusion. His bone marrow examination revealed 7.0% of myeloblasts and showed 15% of recipient cells by STR. Since he had mild renal and cardiac dysfunction and his AML did not progress rapidly, we decided not to give him an intensive chemotherapy. He was given azacitidine (AZA) 75 mg/m^2^ for 5 days following by donor lymphocyte infusion (DLI) on day 6 and 13 after AZA with 0.8 × 10^7^ /kg and 2 × 10^7^ /kg of CD3+ cells from the donor, respectively. He did not develop any grade of GVHD. Since his white blood cells (WBC) and platelets decreased, for his 2nd course, he received decreased dose of AZA (37.5 mg/m^2^) for 5 days following by DLI on day 6 and 13 after AZA with 3 × 10^7^/kg and 5 × 10^7^/kg of CD3+ cells, respectively. After 2 courses of AZA with DLI, his bone marrow still contained 14.4% blasts and STR showed 33.6% of recipient cells. We planned to proceed with a 2nd stem cell transplantation using the same donor PBSC. Meanwhile, he received a third course of AZA without DLI since there was no donor lymphocytes left. We had originally planned for him to receive the conditioning regimen that consisted of fludarabine 25 mg/m^2^ (on day -6 to -2) and melphalan 70 mg/m^2^ (on day -3, and -2) and fractionated GO on day -21, -18, and -15. For prophylaxis against SOS, he was given ursodeoxycholic acid until day 30 and intravenous recombinant thrombomodulin 380 U/kg for 7 days. There was no infusion reaction or any other severe RRT including SOS during GO therapy. However, on day -13, after he received the final dose of GO, he developed right lower abdominal pain with high grade fever. The abdominal CT scan revealed enlargement of appendices with inflammatory changes. He was diagnosed with appendicitis. At this point, his WBC count was 700/μl (Neutrophil 0/μl) and platelets were transfusion dependent. We started administration of broad-spectrum antibiotics to him and decided not to perform surgery because of his bone marrow suppression. Also, we terminated the rest of the patients conditioning regimen and the patient did not receive any further chemotherapeutics. He responded to antibiotics therapy and his condition gradually improved. The bone marrow examination on day -1 revealed hypocellular marrow without myeloblast proliferation. Although we gave him neither fludarabine or melphalan and his bone marrow had started recovering, we still infused peripheral blood stem cells from the donor (CD34+ cells 1.4 × 10^6^/kg) as planned on day 0. The patients appendicitis resolved by day 8. The STR on day -1 showed complete donor chimerism and the WT-1 level has been at undetectable level since 2nd HSCT. The patient became independent from transfusion and has been in CR for 18 months after 2nd PBSCT under maintenance therapy with AZA for 1 year. His clinical course is summarized in Figure 1.

We have obtained written informed consent from the patient for this publication.

## 3 Discussion

Although our patient received GO monotherapy as a conditioning regimen for 2nd HSCT that was outside a plan, it induced sustained remission for more than 18 months under the maintenance therapy with AZA. Our findings suggest that GO therapy may be one solution to improve the dismal prognosis of relapsed AML after allo-HSCT.^[1]^ Although using GO as a part of conditioning regimens for HSCT has been reported^[6–8]^ the majority of transplantation physicians usually avoid using it. One possible reason likely arises from safety concerns, especially regarding potential development of SOS in patients receiving 9 mg/m^2^ of GO within 3 months of standard conditioning regimens.^[9,10]^ Additionally, GO was once withdrawn from the commercial market (except in Japan) in 2010 because clinical trials could not confirm benefit and demonstrated safety concerns.^[11]^ However, after several studies showed promising results with fractionated dosage of GO,^[4,5]^ and with increasing understanding of GO dosing, in 2017, GO has returned to market in multiple other countries. Also recently, Ho et al reported that prior GO exposure in allo-HSCT was not associated with an increased risk of post-transplant SOS.^[12]^

There are also several case reports of GO monotherapy with or without DLI for relapsed AML after allo-HSCT, mostly from Japan^[13–17]^ (Table 1). Since GO therapy should not suppress graft-vs-leukemia effect by cytotoxic T cells,^[16]^ it might be a suitable therapy for relapsed AML after allo-HSCT. All reported cases showed sustained CR without additional cytotoxic therapies. To be precise, our case was not treated by GO monotherapy, but with PBSC infusion. However, similar to these reported cases, GO itself played the main roll of inducing remission in this patient. This indicates GO monotherapy may be sufficient to induce CR in some cases with low tumor burden including our case.

**Table 1:**
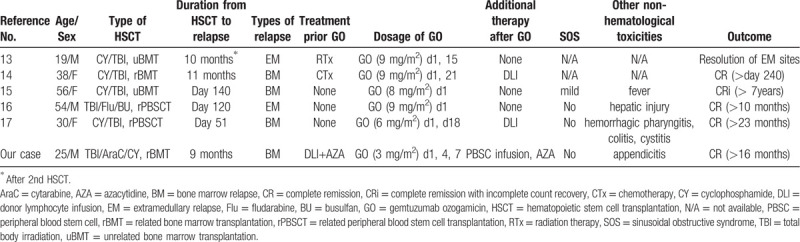
Summary of the cases who received GO monotherapy for relapse AML after allo-HSCT.

In summary, GO may be an effective treatment for relapsed AML after allo-HSCT as either monotherapy or a part of conditioning regimen. Further controlled studies will be necessary to define the optimal dosage of GO.

## Acknowledgments

The authors thank Dr. Thomas Shum for critical review of the manuscript.

## Author contributions

RS and HT contributed to the patient treatment, the study concept and wrote the manuscript. SS, TM, TY, and KM contributed to patient care and collected data. HT, JO and NS edited the manuscript. All authors read and approved the final manuscript.
